# Hybrid Agricultural Monitoring System with Detachable, Biodegradable, and Printed pH Sensors with a Recyclable Wireless Sensor Network for Sustainable Sensor Systems

**DOI:** 10.1021/acsaelm.4c02141

**Published:** 2025-03-21

**Authors:** Andrew Rollo, Joseph Cameron, Jose Diego Fernandes Dias, Radosław Cichocki, Beata Synkiewicz-Musialska, Jia Ren, Shoushou Zhang, Jeff Kettle

**Affiliations:** † James Watt School of Engineering, University of Glasgow, Glasgow G12 8QQ, U.K.; ‡ Łukasiewicz Research Network—Institute of Microelectronics and Photonics, Kraków 30-701, Poland; § Electronic Information and Physics College, Central South University of Forestry and Technology, Changsha, Hunan 410004, P. R. China; ∥ Bangor College, Central South University of Forestry and Technology, Changsha, Hunan 410004, P. R. China

**Keywords:** pH sensor, impedance sensor, molybdenum disulfide, digital agriculture, life cycle assessment

## Abstract

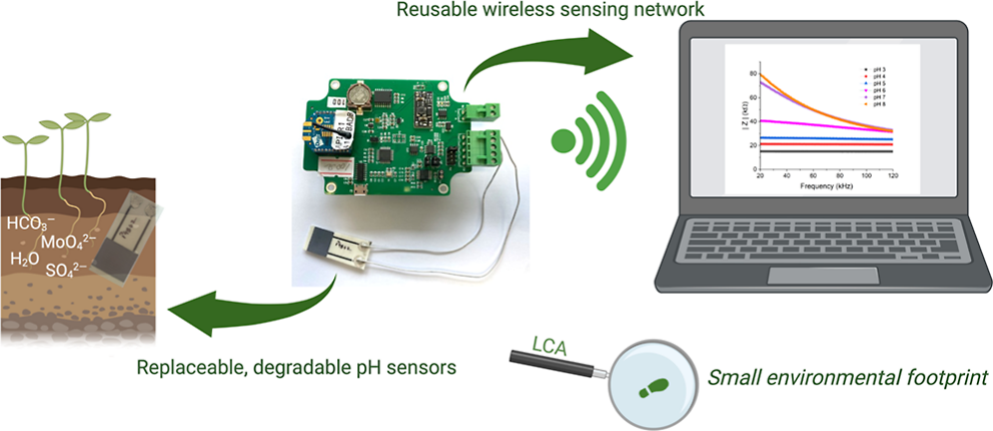

Sustainable food production is one of the key challenges that humanity must overcome to combat global malnutrition and meet the projected increase in the demand for food. Digital agriculture, with the application of sensors to monitor factors such as pH, humidity, and temperature, can improve the efficiency of crop production. However, the sustainability of these devices must be considered. In this work, we report the development of impedance-based pH sensors by using biodegradable materials. It is demonstrated that impedance is an effective way to measure differences in pH using a molybdenum disulfide-based sensor. These sensors can detect agriculturally relevant compounds, as demonstrated by ethephon in this paper, where the active compound’s concentration alters the solution’s pH. We also demonstrate how the molybdenum disulfide pH sensors can be used with our developed wireless sensor network, which can be used for field measurements, giving good agreement compared to impedance measurements using an electrochemical workstation. Life cycle assessment analysis shows that combining a recyclable wireless sensor network with replaceable and degradable sensors leads to a small environmental footprint. As such, this is a promising approach to digital agriculture, which can contribute to more sustainable food production while minimizing the level of electronic waste generation.

## 1 Introduction

Sustainable food production is one of the most crucial challenges that humanity is currently facing. As of 2023, approximately 10% of the global population, numbering over 800 million individuals, endures chronic malnutrition.^1^ Additionally, the demand for food is projected to increase substantially, with estimates indicating a 70% increase in total food consumption by 2050, driven by population growth and rising income levels.^2^ To combat this crisis, investigating and implementing comprehensive strategies to augment food production sustainably is essential. Enhancing crop yield efficiency is one way that the increasing demand for foodstuff production can be confronted. Digital agriculture is an approach that has the potential to obtain results.^3−6^ This approach involves adopting technologies that include Internet of Things (IoT) devices capable of monitoring conditions in real-time and requires a new generation of cost-effective and sustainable sensors that have the capacity to accurately track parameters such as temperature, humidity, nutrient concentration, and pH.^3^ The development and deployment of these technologies have an environmental impact at each stage of their life cycle as the underpinning ICT hardware is often made with unsustainable materials and processes. Furthermore, at the end-of-life cycle, most of the electronic hardware (up to 80%) ends up as electronic waste (e-waste), which poses environmental and public health risks.^1,7^ Digital agriculture provides a unique opportunity to evaluate the use of ICT in an impactful real-life application, while providing the necessary testbed to evaluate the new sensors based on ‘degradable’ or ‘compostable’ materials to reduce the amount of e-waste generated.^8^ The development of degradable or transient devices has been advocated in recent years by adopting resource efficient printing processes along with using bioderived or naturally degradable electronic materials as a means to create zero waste. Such materials can even act as biofertilizers and soil amendments at the end-of-life (EoL).^9^ For instance, biodegradable electronic systems such as chitosan-based devices have been shown to offer an environmentally friendly alternative while also being permeable and degradable.^10,11^

In particular, accurate measurements and monitoring of pH are essential for optimizing crop growth, nutrient uptake, and yield.^12^ The most popular type of printed pH sensor is a potentiometric sensor, consisting of a reference (or quasi-reference) electrode, a counter electrode, and a modified working electrode. The working electrode is modified with a material which has a surface that can be modified in the presence of H^+^ or HO^–^ ions, which in turns alters the energy levels of the modified electrode, causing a change in potential. Some of the best-performing and most stable potentiometric sensors are fabricated using oxides of platinum group metals such as IrO_2_^13,14^ or RuO_2_.^15,16^ However, there is a pressing need to replace such materials due to the harmful environmental impact of extracting and purifying these metals, which are considered to be “critical rare metals” (CRMs). Moreover, these materials, as well as silver-based electrodes, are not biodegradable, meaning that to improve the sustainability of such sensors, recycling processes must be carried out.

An alternative approach that can be taken is to use sensors where the impedance of the sensor changes at different pH. These sensors are simpler, with only a substrate, interdigitated electrodes (IDEs), and an acid/base sensitive active material (e.g., ZnO^17^). Such sensors can be fabricated using low-energy solution processing methods, such as screen printing, and avoid the need for a Ag/AgCl reference electrode. Silver is not a biodegradable element, and printed silver electrodes have been shown to have a high environmental cost;^18^ therefore, impedance-based pH sensors can be fabricated more sustainably in comparison to potentiometric sensors.

Impedance-based sensors offer several advantages, including simpler fabrication and lower environmental impact due to the absence of nonbiodegradable materials such as Ag/AgCl and the need for a reference electrode. However, these benefits come at a cost, where unlike potentiometric sensors, impedance measurements can be sensitive to environmental noise and variations in setup conditions. Additionally, their calibration may be less robust than that of potentiometric devices that rely on well-established reference electrodes for absolute pH measurements.^17,19−22^

In this paper, we present the use of impedance-based pH sensors with a detachable biodegradable front-end sensor and reusable electronics hardware unit. The sensors were fabricated onto a biodegradable substrate, and low-energy screen-printing processes were used for the deposition of electrodes and the sensing layer. We have made a conscious choice of materials for the active layer, electrode, and substrate by considering the degradability of materials at end-of-life, focusing on using elements that can be taken up as nutrients by plants.^23^

All macro- and micronutrients were analyzed (see Table S1) and matched against the literature of pH-sensitive materials. To combine performance in pH sensor and biodegradability, molybdenum disulfide (MoS_2_) was selected as the sensing layer and graphene-carbon (G-C) ink for conductive tracks. These materials were selected as they can contribute positively to soil health by increasing nutrient availability and supporting the growth of microorganisms. Sulfur plays a critical role in producing proteins and other organic compounds. The ideal range of sulfur concentration in soil is between 17 and 397 μg/g. On the other hand, molybdenum is required in trace amounts of approximately 0.00001%. It is essential for enzyme activity and nitrogen fixation in legumes. Both these nutrients are vital for maintaining optimal plant health and productivity. Therefore, it is crucial to adopt informed agronomic strategies for balanced nutrient management.^23−27^

Previous studies on the degradation of molybdenum disulfide have shown that the biodegradation occurs through reaction with oxygen to form the molybdate and sulfate ions^28−31^ with possible reaction stoichiometries shown in eqs 1 and 2.
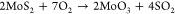
1
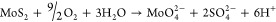
2

Kurapati and co-workers studied the degradation of molybdenum disulfide in hydrogen peroxide as well as in the presence of horseradish peroxidase and human myeloperoxidase enzymes. X-ray photoelectron spectroscopy showed that there was oxidation from Mo(IV) to Mo(VI), indicating that either MoO_3_ or MoO_4_^2–^ had been formed.^30^ Similarly, the analysis of air-saturated, water-based suspensions of MoS_2_ using inductively coupled plasma atomic emission spectroscopy by Wang et al.^29^ showed an increase in the concentration of water-soluble MoO_4_^2–^ over time, with a change in the pH of the solution consistent with the degradation mechanism shown in eq 2. Given that MoO_4_^2–^ and SO_4_^2–^ are the nutrient form taken up by plants (Table 1, with more detail provided in Table S1), the biodegradation of MoS_2_-based sensors can be considered beneficial for agricultural applications.

**Table 1:**
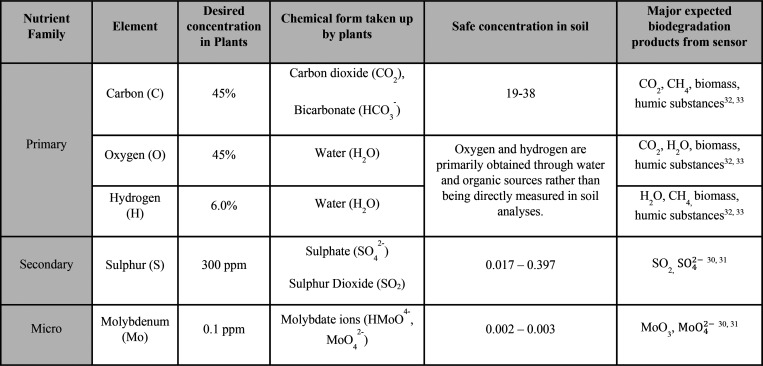
Nutrients Absorbed by Plants from the Sensor’s Biodegradation

In addition to the development of the biodegradable sensor, we show how the sensor can be incorporated into a novel wireless sensing network, demonstrating the potential to perform pH monitoring in the field without the need for expensive and complex equipment. A summary of different properties of IoT technologies is listed in Table S2. This work builds on previous efforts in wireless sensor networks, which have been used for energy harvesting^34,35^ and potentiometric sensors,^36,37^ for example, extending their application to biodegradable impedance sensors for precision agriculture. By using a detachable biodegradable sensor with a reusable electronics module. We demonstrate how our novel sensors and wireless sensing network can be used to monitor substances relevant to agriculture. We used the sensor and wireless network to demonstrate the ability to detect ethephon. (2-Chloroethyl)phosphonic acid, known as ethephon, is the most widely used plant growth regulator in the world, yet it is toxic; therefore, it is important to be able to monitor this compound when used for fruits or vegetables for consumption or in groundwater, for example. Finally, we show how using a biodegradable front-end sensor and recyclable electronics hardware unit is a sustainable way of operating IoT systems by quantifying with life cycle assessment (LCA) approaches. Our approach shows that using detachable sensors in conjunction with a reusable electronics module is a better method to operate agritech WSNs from a sustainability perspective.

## 2 Methods

### 2.1 Sensor Fabrication

An Aurel C920 screen stencil printer was used to print the layers used in the sensors. IDEs (54 mm width, 0.5 mm spacing, and 10 μm thickness) were printed onto a poly(3-hydroxybutyrate-*co*-3-hydroxyvalerate) (PHBV) (0.5 mm thick, Goodfellow) through a stencil using a G-C paste (Sun Chemicals, C2171023D1) before being dried at 100 °C for 1 h. The screen printer is able to produce line widths of approximately 100 μm using G-C paste; further tests could be conducted in order to test the resolution with smaller mask sets. The sheet resistance was determined to be 10 Ω/sq, measured by using a four-point probe (Ossila). The ink used to deposit the MoS_2_ layer was prepared using 40 wt % MoS_2_ (99% nanopowder, 90 nm diameter, Sigma-Aldrich) in a binder solution (8 wt % ethyl cellulose in terpineol), according to a previously reported procedure.^38^ The ink was screen printed over the G-C IDEs before being dried in an oven at 100 °C for 1 h; an image of a typical sensor is given in Figure 3b.

### 2.2 Sensor Characterization

The MoS_2_ layer was characterized using Raman spectroscopy with a Renishaw inVia InSpect confocal Raman microscope with a 514 nm laser. Spectra were collected for a 3 × 4 grid, with 30 μm separating the points. Scanning electron microscopy (Hitachi SU8240) was used to characterize the surface morphology of the active layer.

Electrochemical impedance spectroscopy was carried out using a Metrohm Autolab electrochemistry station over a range of 10 Hz–1 MHz at a rate of 10 points per decade and a rms voltage of 10 mV. The sensors were tested by ensuring the MoS_2_ layer was fully immersed in solution.

The sensors were calibrated in solutions of pH 3 – 8, the solutions were prepared using hydrochloric acid (HCl) or sodium hydroxide (NaOH). The pH of each solution was verified using a calibrated pH meter (Hanna Instruments). Sensors were washed in a solution of the corresponding pH before being immersed in a separate solution of the same pH and left for two min before testing. For studies using ethephon, a range of aqueous solutions from 10^–3^ M to 5 × 10^–7^ M was prepared, and impedance measurements were carried out as described above.

### 2.3 Wireless Sensing Network: Design and Fabrication

**Figure 1:**
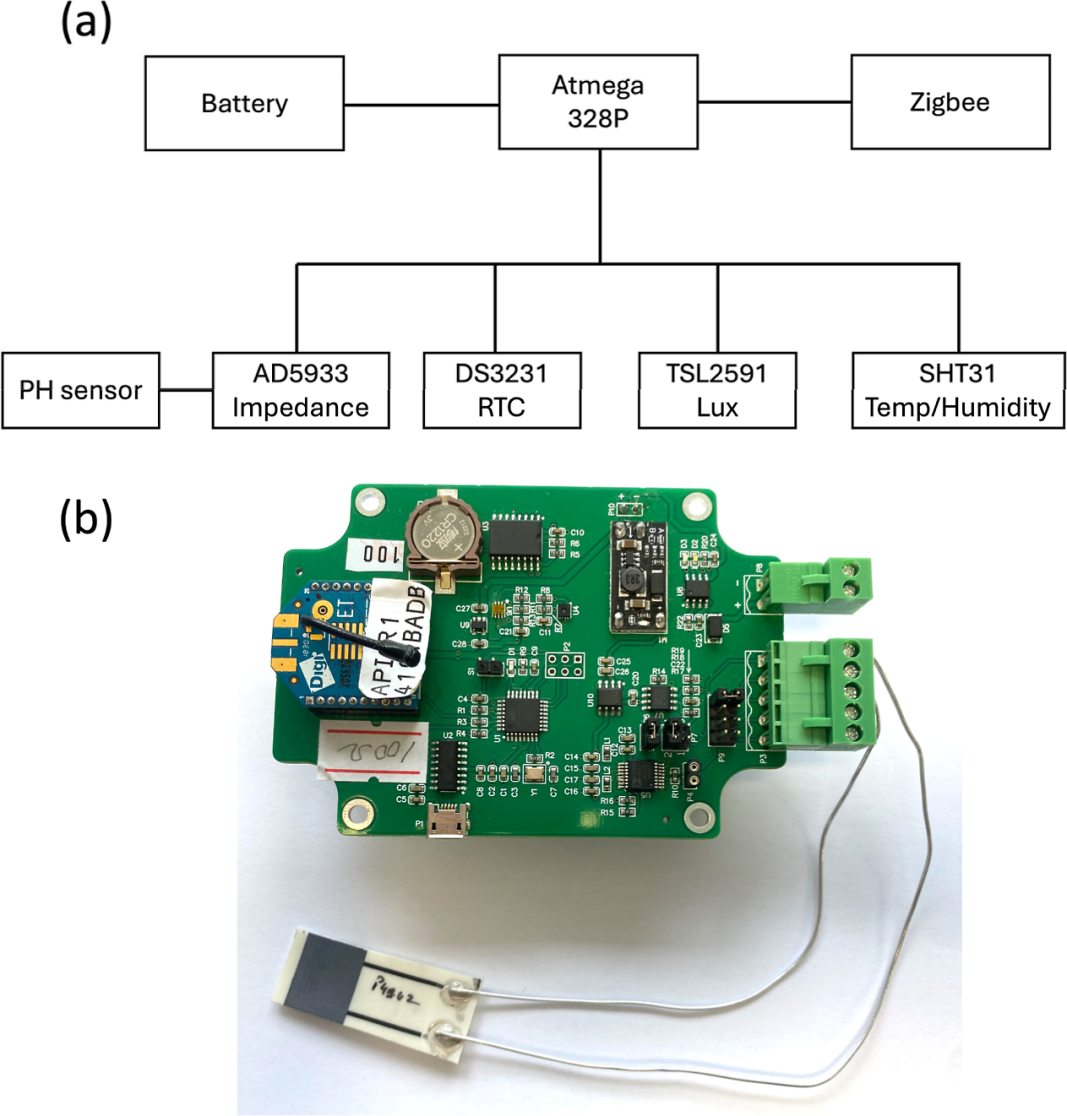
(a) Block diagram of the components of wireless sensing network and (b) image of the WSN with a sensor attached.

Figure 1 illustrates the design and configuration of the recyclable wireless sensor network (WSN). As sensors degrade, the central electronics can be recycled in further measurement campaigns. The unit is inspired by a previous module developed by the authors,^34^ with the addition of impedance measurement capability for detachable pH sensors and some changes to the sensors and energy management module. The new system specifications are listed below, while comprehensive details are listed in the Supporting Information.

In Figure 1a the block diagram outlines the WSN node’s key components, including the ATmega644PA microcontroller (MCU) and several integrated sensors: the SHT31 for temperature and humidity (replacing BME280 in the previously reported system), the TSL2591 for light levels,^34^ the AD5933 for impedance measurements,^39^ and the DS3231 real-time clock (RTC).^40^ The DS3231 RTC ensures accurate timing for synchronized data collection through its temperature-compensated crystal oscillator. Figure 1b shows an image of the WSN with an attached pH sensor through zinc wires.

The sensor node was constructed with an AD5933 impedance converter, which is capable of impedance analysis and a new addition to the wireless sensing network. The AD5933 is a 12 bit, 1 MSPS analog-to-digital converter (ADC) and a 16 MHz frequency generator, all integrated into the chip. This IC was chosen due to its ability to measure the complex impedance including the values of both magnitude and phase. Data communication, which employs a ZigBee RF module, facilitates the wireless transmission of collected data. Deploying multiple sensor nodes equipped with the AD5933 enables the monitoring of a system simultaneously at multiple points. This capability is vital in applications requiring comprehensive coverage or transmitting data for offsite analysis.

An important aspect of the design was to minimize the power consumption, so that the node could be powered by an OPV module under indoor lighting levels, maintaining the battery charge level and ensuring long-term and autonomous operation. Although the node can consume approximately 370 mW when it is actively transmitting data, it requires about 1 mW when it is in sleep mode and 15 mW when sampling data from the impedance node. Therefore, the energy consumption largely depends on transmission rate, i.e., how regularly data is transmitted from the WSN. Assuming transmissions every 4 h, the WSN consumes 14.3 mW h of energy per day. This was achieved by using sensors capable of low power sleep modes, an IC load switch to turn off the impedance sensor, and putting both the ZigBee and MCU into sleep mode between measurements. The only component that stayed awake was the RTC, which toggled a hardware interrupt on the MCU after a set period had elapsed, allowing the next measurement cycle to be initiated.

The communication requirements for these nodes are for a small amount of data to be sent at 15 min intervals from sensing locations. Such time intervals enable data collection during extreme events to be collected (e.g., torrential rain) as well as long-term monitoring. There are several technologies and protocols available for wireless low-power, wide-area networks, but in this case, the node uses a ZigBee RF module to transmit the sensor measurements back to a central datalogger via a star network configuration. ZigBee has the advantages of easy deployment, ability to form flexible mesh networks, and a high data rate. The relatively low transmission range (50–200 m) was not a problem in the environment being monitored. In this node, a Digi XBEE S2C 802.15.4.RF ZigBee radio module has been used. The module can be put into sleep mode, which keeps the power requirements down. The PCB was designed so that it could be fabricated with either a U.FL or an SMA RF socket, allowing a choice of aerials to be attached. When the node is due to take its next set of measurements, the RTC will poll the interrupt pin on the MCU to wake it up. The MCU will then power the AD5933 circuitry and wake up the Zigbee module. The MCU will process through AD5933 for the desired experiment. The MCU will then retrieve the measured data from the AD5933 IC before the sensing circuitry. Having taken all the measurements, the MCU will build the payload message with the results, transmit it over ZigBee, and then put the ZigBee module to sleep. Finally, the MCU will program the RTC with the next wake-up time and put itself to sleep, ready for the next measurement cycle.

In order to assess the performance of the WSN compared to lab-based electrochemistry systems, the accuracy of the WSN was determined using the following equation
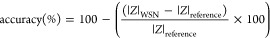
3where |*Z*|_reference_ was the impedance measured using the Autolab electrochemical workstation.

## 3 Results and Discussion

### 3.1 Material Characterization and Metrology

The developed impedance sensors were designed to be fully biodegradable, with the ability to provide nutrients to plants after degradation. We chose molybdenum disulfide as a sensing layer due to the ability of plants to use molybdenum and sulfur. It has previously been used as a pH-sensitive layer in field-effect transistors^41^ and is a suitable material due to its stability and lack of solubility in aqueous solutions. The substrate, PHBV, contains carbon, hydrogen, and oxygen and has previously been shown to be biodegradable,^32^ while G-C was chosen as the electrode material. A summary of the role of the elements present in our sensor as plant nutrients is presented in Table S1.

**Figure 2:**
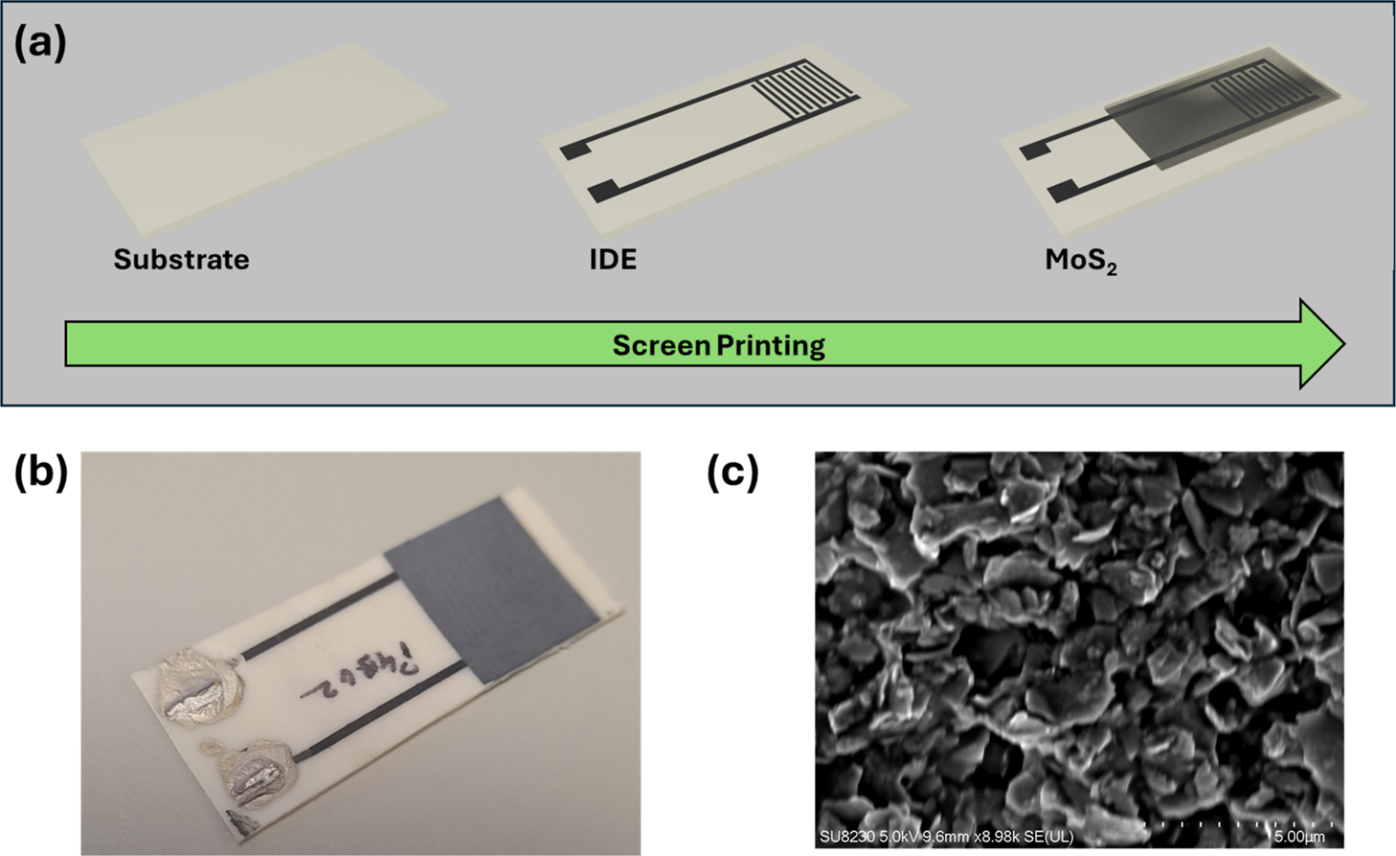
Characterization of the screen-printed sensor device showing the (a) process flow of the two-step screen printing manufacturing process of the device; (b) image of the final printed sensor; and (c) SEM image of the MoS_2_ sensing layer.

Screen printing was used as a low-cost, low-energy method for the deposition of patterned films. Figure 2a provides a representation of the fabrication process for the screen-printed impedance sensor. It begins with the blank substrate, on which the IDE design is printed. After being cured, the printed electrodes are aligned, and a second screen printed layer is added that deposits the MoS_2_ sensing layer. Uniformity in line width and spacing is essential to ensure that the sensor’s response is reproducible and reliable. The printed MoS_2_ layer, with ethyl cellulose binder, gives a uniform, dark gray film (Figure 2b). The Raman spectra (Figure S1) for the MoS_2_ sensing layer exhibit two distinct peaks related to the basic vibrational modes of MoS_2_. The first peak, located at 382.1 cm^–1^, is associated with the E_2g_ band of MoS_2_, which represents the in-plane vibrations of Mo and S atoms where the Mo and S atoms move in the same plane. For the second peak, located at 407.9 cm^–1^, the A_1g_ mode. In the A_1g_ vibrational mode, the S atoms vibrate perpendicular to the Mo atoms that remain stationary. The increase in the intensity of the peak is related to the thickness of the layer, and the sharpness of the peak indicates a high level of crystallinity and purity of the MoS_2_.^42,43^

The SEM image in Figure 2c depicts the microstructural detail of the MoS_2_ sensing layer, which consists of granular flakes with a high aspect ratio. This rough and porous surface is particularly suitable for sensing as it provides a larger surface area for interaction with ions. The sensor’s sensitivity is heavily influenced by its porosity and microstructure, as they directly impact the reaction kinetics of the sensor with the target analyte—the H^+^ or HO^–^ ions in solution—so processing was optimized to give this morphology.

### 3.2 pH Sensor Results

**Figure 3:**
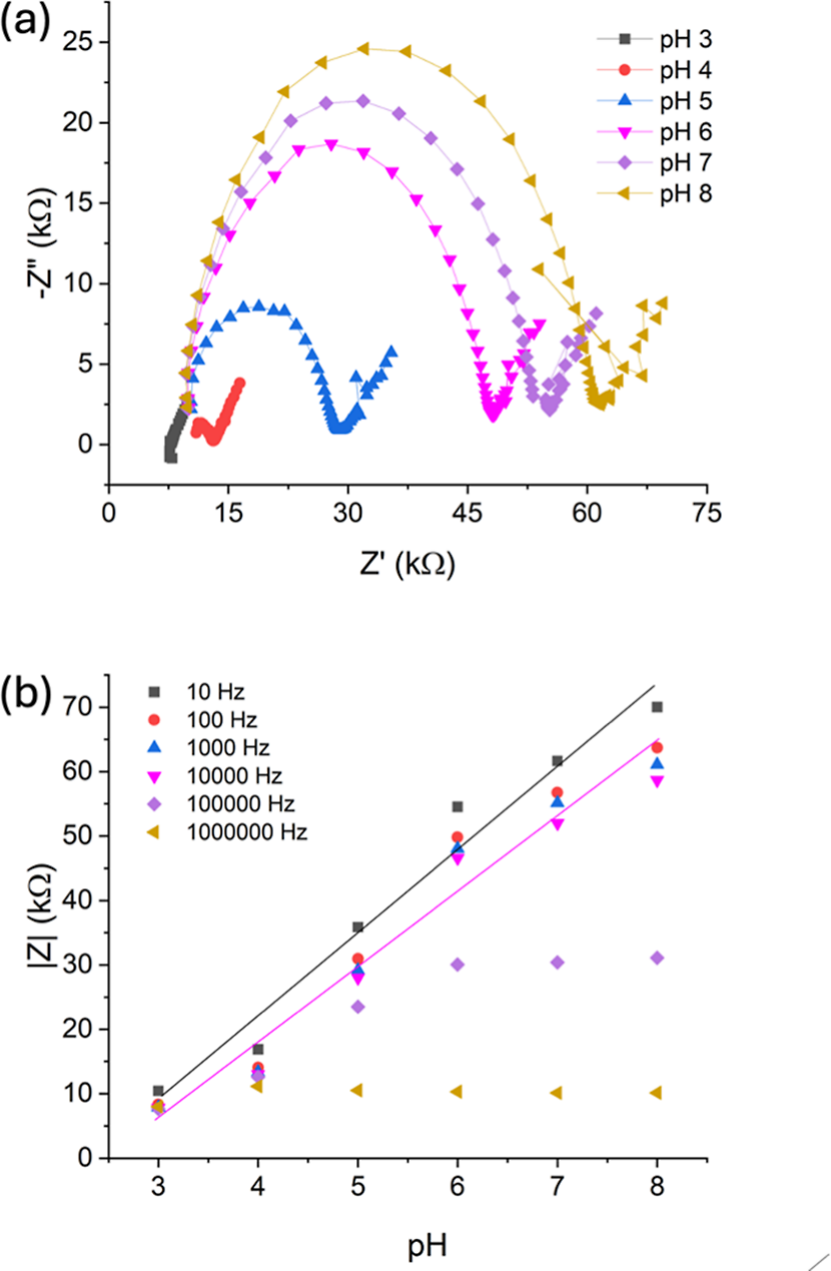
(a) Nyquist plots of PHBV/G-C/MoS_2_ sensor in different pH solutions; (b) modulus of impedance vs pH at different frequencies, with line of best fit shown for measurements at 10 Hz (*R*^2^ = 0.97) and 1000 Hz (*R*^2^ = 0.96).

Electrochemical impedance spectroscopy of the sensors was carried out in solutions of different pH values to determine the sensor response. From the Nyquist plot in Figure 3a and a plot of |*Z*| against pH, it is shown that there is an increase in impedance with increased pH. The trend is linear, with a *R*^2^ value of 0.97, and the sensitivity of the sensor is determined to be 12.9 kW/pH at 10 Hz. The relationship does not hold at high frequencies (100 kHz and 1 MHz), which indicates that the capacitance of the MoS_2_ layer is key to the sensing mechanism.

Equivalent circuits were fitted to the electrochemical impedance spectroscopy data (Figures S2 and S3), with a circuit of a series resistor combined with a resistor in parallel with a constant phase element showing good agreement with the experimental plots. In general, the series resistance is fairly constant, with the sensor at pH 3 and pH 8 showing lower Rs. However, there are noticeable trends with the resistor in parallel, with the Rp increasing with increased pH. This can be attributed to a lower charge transfer resistance of MoS_2_ surface with decrease in pH. MoS_2_ is an n-type semiconductor, therefore the charge transfer is favored with increased concentration of H^+^ ions. The admittance is highest at pH 3, where there is the highest concentration of ions, and decreases with decreasing pH until between pH 6 and 8, where the admittance is similar due to the low concentration of ions present in the solution.

**Figure 4:**
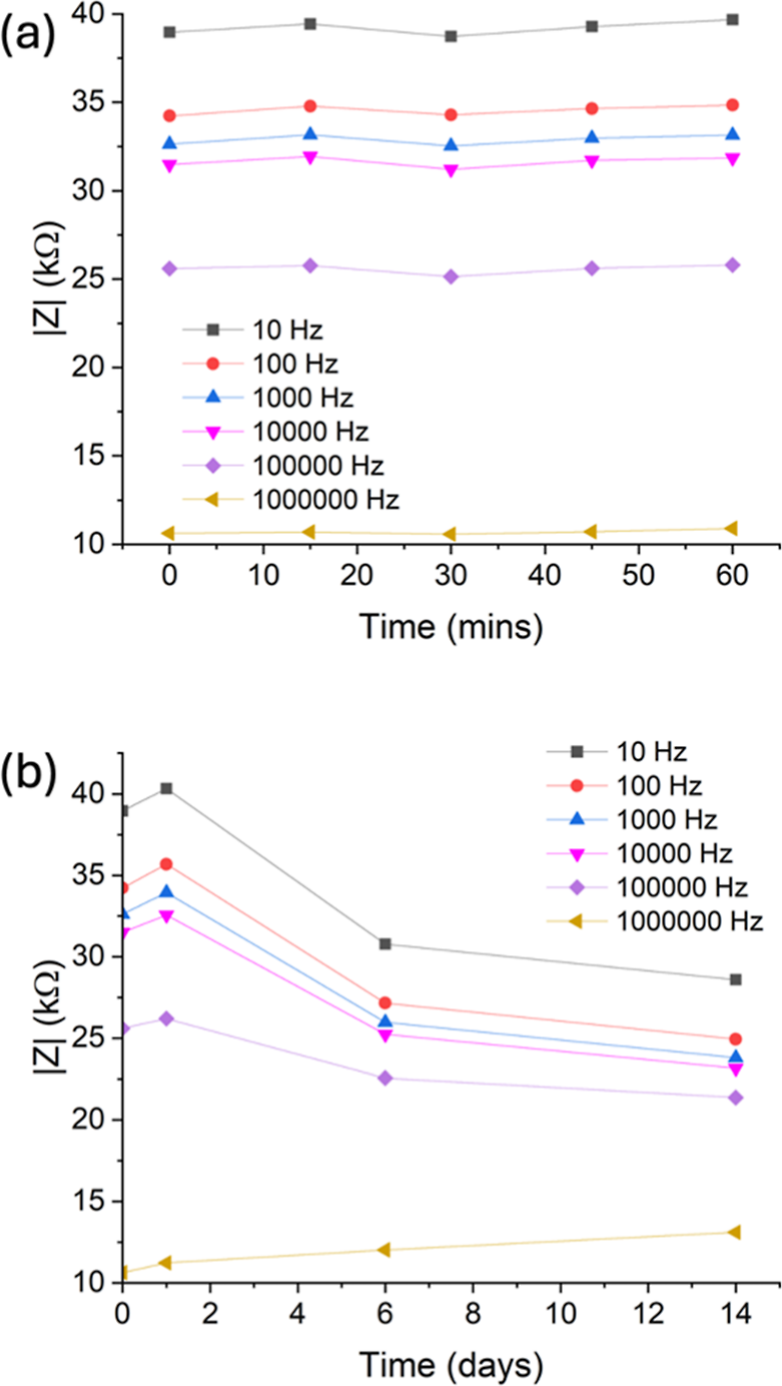
(a) Modulus of impedance at different frequencies over 1 h; (b) modulus of impedance at different frequencies over 2 weeks.

To improve the sustainability of biodegradable sensors, it is important that they demonstrate stability for continuous monitoring rather than being restricted to a single use. Therefore, we studied the stability of the MoS_2_-based sensor in pH 5 solution over time, which is shown in Figure 4. The sensors showed no observable degradation over time in solution, suggesting good adhesion of the MoS2 film on the PHBV substrate. The short-term stability of the sensor is very good, with little deviation in impedance after 1 h. In the longer term, the impedance generally decreases over time, but if compared to the calibration graph in Figure 4b, it can be unambiguously identified that the measurement is carried out in pH 5 solution. This demonstrates that the PHBV/G-C/MoS_2_ sensor shows promise in being applied for continuous pH monitoring.

**Figure 5:**
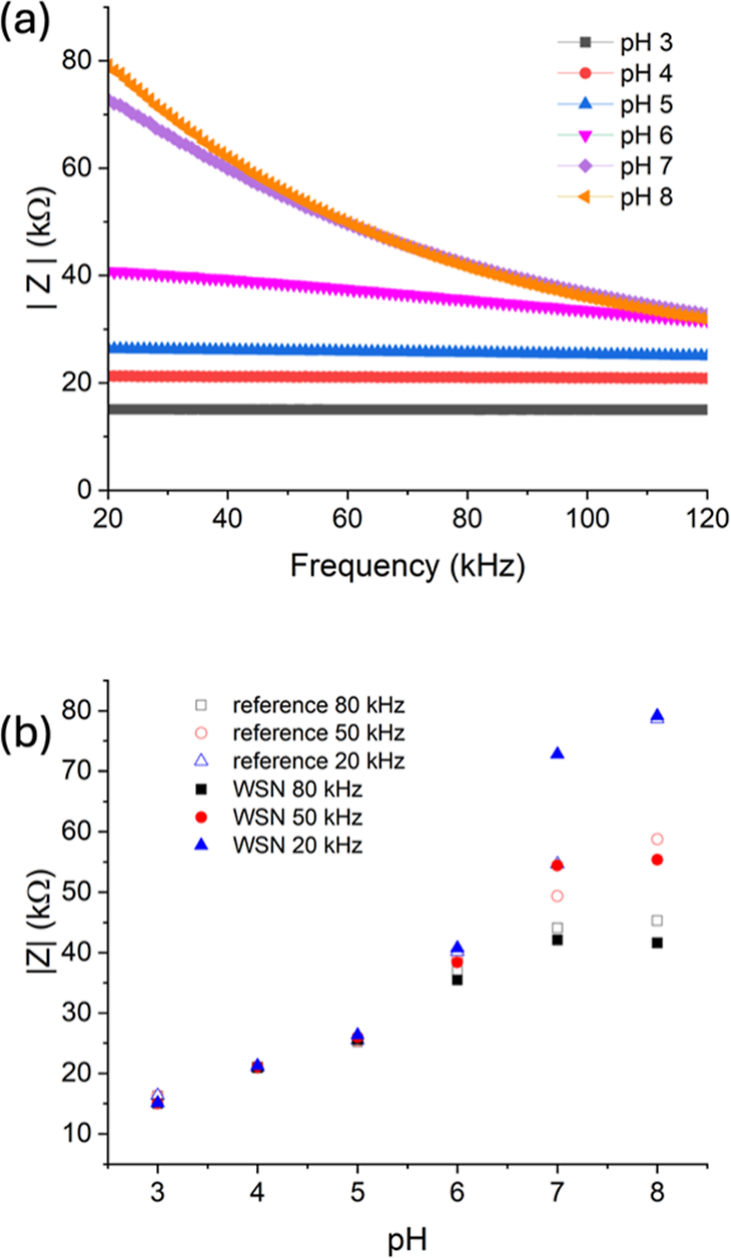
(a) Modulus of impedance vs frequency measured using the WSN; (b) modulus of impedance vs pH measured using the WSN compared to reference measured using an electrochemical workstation.

For sensors to be applicable, it is important that they can be used with portable impedance measurement systems. Therefore, we used our developed WSN with MoS_2_-based sensors to measure the impedance of different pH solutions. This was cross verified with measurements using a Metrohm electrochemical workstation in order to determine the reliability of the WSN. The plots are shown in Figure 5.

The printed sensor used for these experiments has a slightly higher impedance per pH compared to the calibration plot shown in Figure 3b, but overall, there is good agreement, and there is a general linear relationship with impedance modulus and pH. The WSN measures over a frequency range between 20 and 120 kHz; therefore, points at 20, 50, and 80 kHz were taken from data measured using the electrochemical workstation as a comparison (Figure 5b).

The accuracy compared to the electrochemistry equipment as a reference was calculated according to eq 3, as shown in Section 2.3, and a summary of these calculations is shown in Table 2.

**Table 2:** Accuracy of the WSN Compared to the Electrochemical Workstation

	accuracy at 20 kHz (%)	accuracy at 70 kHz (%)	accuracy at 120 kHz (%)
pH 3	100	80	89
pH 5	100	89	88
pH 7	100	89	75

There is good agreement between the two different impedance measurements, which highlights that the WSN can be used to reliably measure the change of impedance in sensors. Most importantly, there is excellent agreement at the lower end of the frequency range, where there is a linear relationship with pH and |*Z*|. Therefore, this system can be considered a promising technology for agricultural sensing, especially where it is crucial to monitor pH changes.

### 3.3 Ethephon Detection

**Figure 6:**
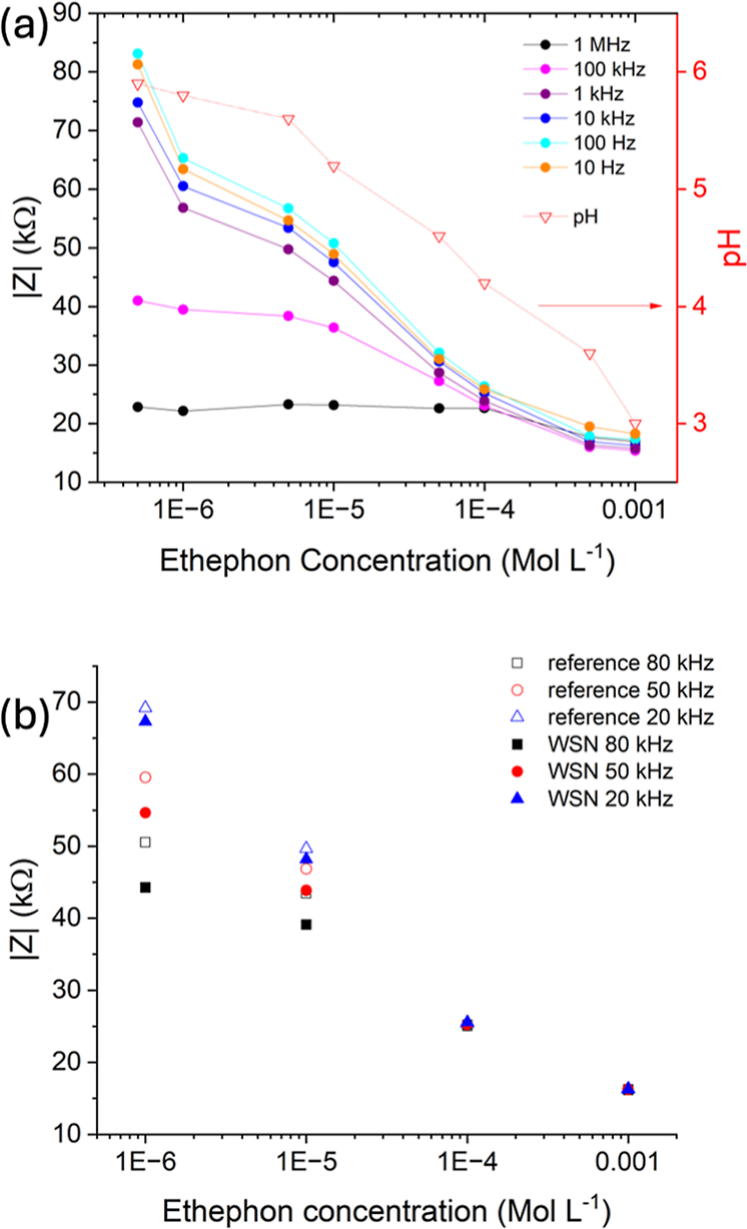
(a) Calibration graph of impedance and pH vs ethephon concentration for PHBV/G-C/MoS_2_ sensor; (b) comparison of impedance measurement of ethephon solutions using the WSN or electrochemical workstation.

Ethephon, a widely used plant growth regulator, is an ecotoxin and harmful to human health. A European Commission report in 2023 identified concerns from use of the substance from the risk to those applying it, consumers, wildlife, and groundwater contamination.^44^ Therefore, a simple sensor to monitor the ethephon concentration would be useful in helping to mitigate some of these risks. Aqueous ethephon solutions are acidic; therefore, we can use our impedance-based pH sensors to detect the concentration of the plant growth regulator, which in turn can inform whether it violates recommended limits in water courses near agricultural sites. A calibration graph is shown in Figure 6a, where the modulus of impedance at different frequencies and pH of solution are plotted against ethephon concentration. The maximum concentration was chosen to be slightly higher than the recommended maximum dilutions required for the use of ethephon for different plants in commercial formulations. Like the initial pH calibration graphs, there is a linear relationship between the log of ethephon concentration and impedance except from 1 MHz and 100 kHz. Having established that the developed sensors were effective in detecting different concentrations of ethephon, we repeated the studies using the WSN to measure the impedance in different solutions. A different sensor, fabricated using the same process, was used for these experiments, and the data was cross verified with measurements using a Metrohm electrochemical workstation. The calibration plot determined using the wireless sensing network is shown in Figure 6b.

The measured impedance shows good consistency with the values measured for the sensor, as shown in Figure 4. There is a linear relationship between the impedance modulus and the concentration of ethephon in the log scale. Like the calibration plot using the WSN in Figure 5b, there is very good consistency in measured impedance from the WSN compared to those determined from the electrochemical workstation. This demonstrates that the WSN with printed PHBV/G-C/MoS_2_ sensors can be used effectively to monitor the concentration of residual ethephon and is well suited for use in the field.

### 3.43.4 Life Cycle Assessment

The sustainability of the hybrid system was evaluated through an LCA using ISO standards 14040, 14044, and 14067. The assessment aimed to analyze the environmental impacts of WSNs and printed sensors, pinpoint environmental hotspots, and compare the impact of reuse of the WSN.

**Figure 7:**
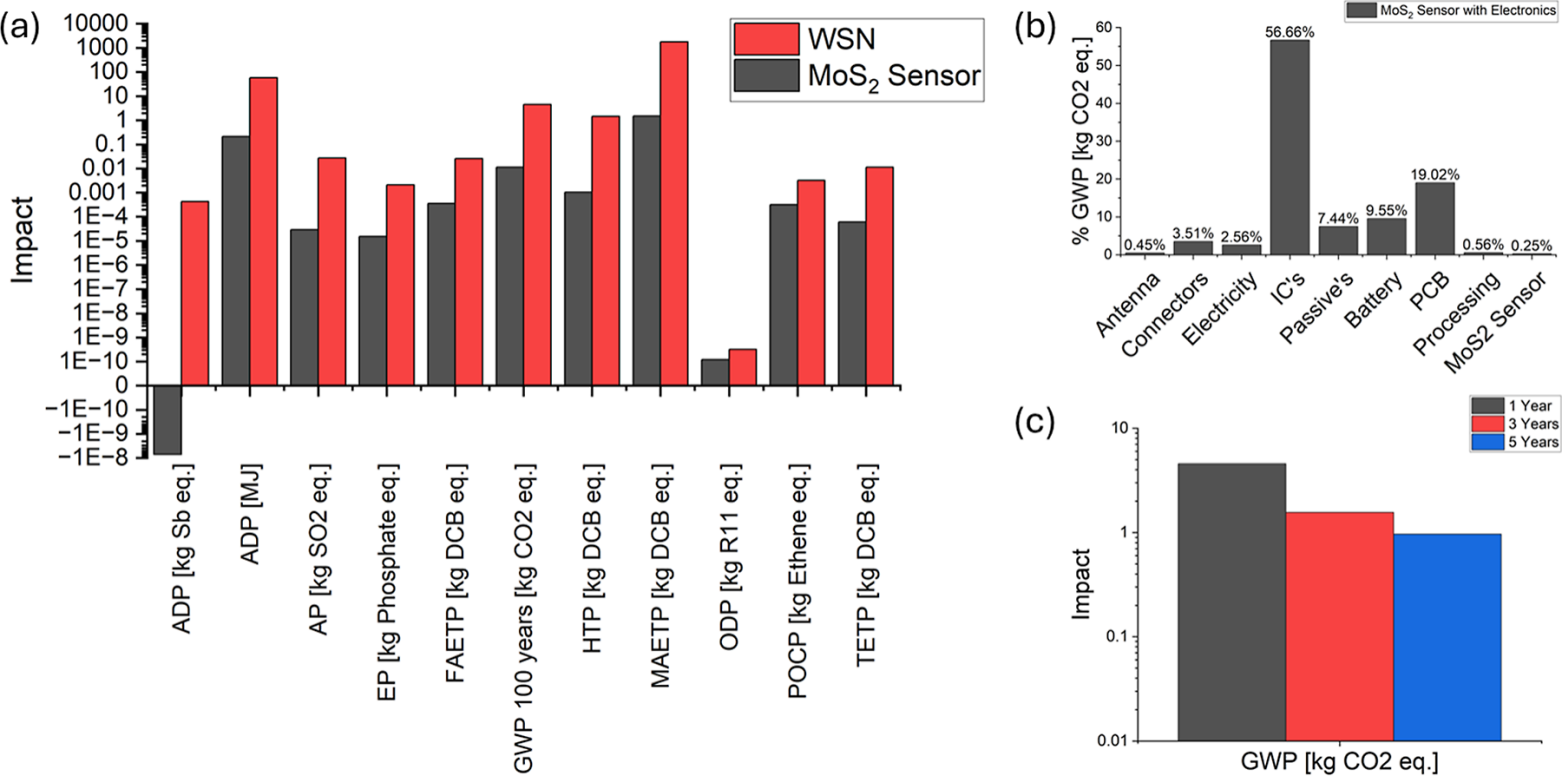
(a) Total environmental impact of the WSN and sensor across various impact categories; (b) percentage breakdown of the GWP by category; and (c) GWP comparison for the system evaluated over 1, 3, and 5 years.

In Figure 7a, the environmental impact of the system is depicted using the “Centrum voor Milieu en Lucht” (CML) benchmark on a logarithmic scale.^45^ The WSN displays higher impacts across environmental categories compared to the MoS_2_ sensor, particularly in the abiotic depletion potential (ADP) elements, where the negative value of the sensor indicates a positive environmental effect in comparison to WSN breakdown. The most significant impacts are in ADP, marine aquatic ecotoxicity potential, global warming potential (GWP), and human toxicity potential. More detailed results can be found in Tables S3 and S4.

Figure 7b presents the breakdown of the GWP for the WSN with a sensor, highlighting the components of the sensor system that have the highest impact on the GWP. The ICs account for 57% of the GWP, while the PCB contributes 18%. This aligns with data on small consumer electronic devices, where the production of ICs and PCBAs has the most significant impact on the entire life cycle. On the other hand, the contribution of the rest of the life cycle phases is lower. Although the sensor has less impact than the electronics, its short lifespan of approximately 3 months in the field necessitates regular replacement, while the WSN can be reused and last for several years without maintenance.

Extending the useful life of the WSN by replacing the degradable sensor 6 times per year can be beneficial, as indicated by the LCA impacts per year shown in Figure 7c. By replacing the sensor, the WSN’s longevity is increased, preventing it from being treated as e-waste. Although extending the device’s lifetime increases the impacts related to the use phase and additional impacts from replacing the sensor, the net benefit, up to 66% and 79% reduction in GWP for 3 year and 5 year lifetimes, respectively, stems primarily from reduced production-related emissions. The total GWP impact decreases as the lifetime increases, thanks to the ability to replace the sensor, which has a significantly smaller impact than the electronics.

## 4 Conclusions

We have demonstrated the fabrication of novel impedance sensors using biodegradable materials. The molybdenum disulfide active layer is sensitive to pH; therefore, when the sensor is immersed in a solution, the measured impedance is directly proportional to the pH of the solution. The sensor shows good stability over 2 weeks in pH 5 solution, which shows promise for stable performance of a biodegradable substrate. In addition to sensor development, we have reported a new wireless sensing network for impedance measurements that shows consistent performance compared to more complex, expensive, and bulky electrochemical workstations. Having established the ability to sense pH, it was demonstrated that the combination of a WSN with an impedance sensor could be used to detect ethephon, the most widely used plant growth regulator in the world, which is harmful to humans and wildlife and is a risk for contaminating groundwater. Therefore, we have demonstrated the use of new biodegradable sensors with portable, recyclable wireless sensing network sensing an agriculturally relevant compound. The LCA interpretation of the system makes a strong case for applying WSN systems with a high environmental impact with low-impact sensors that can be replaced to extend the life of the system and provide accurate and reliable data.

In the coming decades, meeting our required food production will be one of humanity’s greatest challenges. This may involve increased use of a plant growth regulator. Although the most popular compound for this is toxic, we have developed a system that can be used to help avoid the harmful effects of this compounds. However, we anticipate that the wireless sensing network with biodegradable PHBV/G-C/MoS_2_ sensors could also be used more generally, for precision agriculture practices, for example. Monitoring pH can help improve the yield and efficiency of plant growth, and by using biodegradable sensors and reusable WSNs, we have shown that the challenge of increased food production can be tackled without compromising the need to reduce carbon dioxide emissions or e-waste.

## Data Availability

Supporting data associated with this manuscript can be accessed at https://doi.org/10.5525/gla.researchdata.1699.

## Supporting Information Available

Information on elemental nutrients for plants present in the sensors; list of IoT technologies and their power outputs/range; equivalent circuit fits for Nyquist plots of the MoS_2_ sensors in different pH solutions; detailed breakdown of environmental impacts from LCA; and expanded description of the WSN construction (PDF)

## References

[ref1] Forti V., Balde C. P., Kuehr R., Bel G. The Global E-Waste Monitor 2020: Quantities, Flows and the Circular Economy Potential; United Nations University/United Nations Institute for Training and Research, International Telecommunication Union, and International Solid Waste Association: Bonn, Geneva and Rotterdam, 2020. https://collections.unu.edu/eserv/UNU:7737/GEM_2020_def_july1.pdf (accessed 23/01/2025).

[ref2] Rahut D. B.; Aryal J. P.; Manchanda N.; Sonobe T. Chapter 6 - Expectations for household food security in the coming decades: A global scenario. In Future Foods; Bhat R., Ed.; Academic Press, 2022; pp 107–131. 10.1016/B978-0-323-91001-9.00002-5.

[ref3] Sishodia R. P.; Ray R. L.; Singh S. K. Applications of Remote Sensing in Precision Agriculture: A Review. Remote Sens. 2020, 12 (19), 3136 10.3390/rs12193136.

[ref4] Mutunga T.; Sinanovic S.; Harrison C. S. Integrating Wireless Remote Sensing and Sensors for Monitoring Pesticide Pollution in Surface and Groundwater. Sensors 2024, 24 (10), 3191 10.3390/s24103191. 38794044 PMC11125874

[ref5] Liu Y.; Ma X.; Shu L.; Hancke G. P.; Abu-Mahfouz A. M. From Industry 4.0 to Agriculture 4.0: Current Status, Enabling Technologies, and Research Challenges. IEEE Trans. Industr. Inform. 2021, 17 (6), 4322–4334. 10.1109/TII.2020.3003910.

[ref6] Javaid M.; Haleem A.; Singh R. P.; Suman R. Enhancing smart farming through the applications of Agriculture 4.0 technologies. Int. J. Intell. Netw. 2022, 3, 150–164. 10.1016/j.ijin.2022.09.004.

[ref7] Ahirwar R.; Tripathi A. K. E-waste management: A review of recycling process, environmental and occupational health hazards, and potential solutions. Environ. Nanotechnol. Monit. Manag. 2021, 15, 100409 10.1016/j.enmm.2020.100409.

[ref8] Swathi B.; Yamini G.; Sasikala R.; Teresa M.; Jagadeesan D.; Yasa V. R. Sustainable E-Waste Reduction Methods and Models. In Sustainable Solutions for E-Waste and Development, K. V, R. K.; Kannan H., Spodarets D., Khan P. A., Pradhan B. K., Eds.; IGI Global: Hershey, PA, USA, 2024; pp 239–253. 10.4018/979-8-3693-1018-2.ch016.

[ref9] Hankel A.; Heimeriks G.; Lago P. A Systematic Literature Review of the Factors of Influence on the Environmental Impact of ICT. Technologies 2018, 6 (3), 85 10.3390/technologies6030085.

[ref10] Peng X.; Dong K.; Zhang Y.; Wang L.; Wei C.; Lv T.; Wang Z. L.; Wu Z. Sweat-Permeable, Biodegradable, Transparent and Self-powered Chitosan-Based Electronic Skin with Ultrathin Elastic Gold Nanofibers. Adv. Funct. Mater. 2022, 32 (20), 2112241 10.1002/adfm.202112241.

[ref11] Peng X.; Dong K.; Wu Z.; Wang J.; Wang Z. L. A review on emerging biodegradable polymers for environmentally benign transient electronic skins. J. Mater. Sci. 2021, 56 (30), 16765–16789. 10.1007/s10853-021-06323-0.

[ref12] Hartemink A. E.; Barrow N. J. Soil pH - nutrient relationships: the diagram. Plant Soil 2023, 486 (1), 209–215. 10.1007/s11104-022-05861-z.

[ref13] Wang M.; Yao S.; Madou M. A long-term stable iridium oxide pH electrode. Sens. Actuators, B 2002, 81 (2), 313–315. 10.1016/S0925-4005(01)00972-8.

[ref14] Huang W.-D.; Cao H.; Deb S.; Chiao M.; Chiao J. C. A flexible pH sensor based on the iridium oxide sensing film. Sens. Actuators, A 2011, 169 (1), 1–11. 10.1016/j.sna.2011.05.016.

[ref15] Tanumihardja E.; Olthuis W.; Van den Berg A. Ruthenium Oxide Nanorods as Potentiometric pH Sensor for Organs-On-Chip Purposes. Sensors 2018, 18 (9), 2901 10.3390/s18092901. 30200489 PMC6163346

[ref16] Liao Y.-H.; Chou J.-C. Preparation and characteristics of ruthenium dioxide for pH array sensors with real-time measurement system. Sens. Actuators, B 2008, 128 (2), 603–612. 10.1016/j.snb.2007.07.023.

[ref17] Aliyana A. K.; Ganguly P.; Beniwal A.; Kumar S. K. N.; Dahiya R. Disposable pH Sensor on Paper Using Screen-Printed Graphene-Carbon Ink Modified Zinc Oxide Nanoparticles. IEEE Sens. J. 2022, 22 (21), 21049–21056. 10.1109/JSEN.2022.3206212.

[ref18] Naji Nassajfar M.; Välimäki M.; Hakola L.; Eiroma K.; Immonen K.; Abdulkareem M.; Horttanainen M. The effect of conductive ink alternation on the sustainability and functioning of printed electronics. Flexible Printed Electron. 2023, 8 (2), 025015 10.1088/2058-8585/acd650.

[ref19] Manjakkal L.; Sakthivel B.; Gopalakrishnan N.; Dahiya R. Printed flexible electrochemical pH sensors based on CuO nanorods. Sens. Actuators, B 2018, 263, 50–58. 10.1016/j.snb.2018.02.092.

[ref20] Manjakkal L.; Djurdjic E.; Cvejin K.; Kulawik J.; Zaraska K.; Szwagierczak D. Electrochemical Impedance Spectroscopic Analysis of RuO2 Based Thick Film pH Sensors. Electrochim. Acta 2015, 168 (04.048), 246–255. 10.1016/j.electacta.2015.04.048. 27282750

[ref21] Arshak K.; Gill E.; Arshak A.; Korostynska O. Investigation of tin oxides as sensing layers in conductimetric interdigitated pH sensors. Sens. Actuators, B 2007, 127 (1), 42–53. 10.1016/j.snb.2007.07.014.

[ref22] Gill E.; Arshak K.; Arshak A.; Korostynska O. Mixed metal oxide films as pH sensing materials. Microsyst. Technol. 2008, 14 (4), 499–507. 10.1007/s00542-007-0435-9.

[ref23] Hawkesford M. J.; De Kok L. J. Managing sulphur metabolism in plants. Plant, Cell Environ. 2006, 29 (3), 382–395. 10.1111/j.1365-3040.2005.01470.x. 17080593

[ref24] Wang S. P.; Wang Y. F.; Chen Z. Z.; Schnug E.; Haneklaus S. Sulphur concentration of soils and plants and its requirement for ruminants in the Inner Mongolia steppe of China. Grass Forage Sci. 2001, 56 (4), 418–422. 10.1046/j.1365-2494.2001.00285.x.

[ref25] Kaiser B. N.; Gridley K. L.; Ngaire Brady J.; Phillips T.; Tyerman S. D. The Role of Molybdenum in Agricultural Plant Production. Ann. Bot. 2005, 96 (5), 745–754. 10.1093/aob/mci226. 16033776 PMC4247040

[ref26] McGrath S. P.; Micó C.; Zhao F. J.; Stroud J. L.; Zhang H.; Fozard S. Predicting molybdenum toxicity to higher plants: Estimation of toxicity threshold values. Environ. Pollut. 2010, 158 (10), 3085–3094. 10.1016/j.envpol.2010.06.030. 20656390

[ref27] Gupta U. C. Molybdenum in Agriculture; Cambridge University Press: Cambridge, 1997; . 10.1017/CBO9780511574689.

[ref28] Chen X.; Park Y. J.; Kang M.; Kang S.-K.; Koo J.; Shinde S. M.; Shin J.; Jeon S.; Park G.; Yan Y.; MacEwan M. R.; Ray W. Z.; Lee K.-M.; Rogers J. A.; Ahn J.-H. CVD-grown monolayer MoS2 in bioabsorbable electronics and biosensors. Nat. Commun. 2018, 9 (1), 1690 10.1038/s41467-018-03956-9. 29703901 PMC5924366

[ref29] Wang Z.; von dem Bussche A.; Qiu Y.; Valentin T. M.; Gion K.; Kane A. B.; Hurt R. H. Chemical Dissolution Pathways of MoS2 Nanosheets in Biological and Environmental Media. Environ. Sci. Technol. 2016, 50 (13), 7208–7217. 10.1021/acs.est.6b01881. 27267956 PMC5217159

[ref30] Kurapati R.; Muzi L.; de Garibay A. P. R.; Russier J.; Voiry D.; Vacchi I. A.; Chhowalla M.; Bianco A. Enzymatic Biodegradability of Pristine and Functionalized Transition Metal Dichalcogenide MoS2 Nanosheets. Adv. Funct. Mater. 2017, 27 (7), 1605176 10.1002/adfm.201605176.

[ref31] Chen X.; Shinde S. M.; Dhakal K. P.; Lee S. W.; Kim H.; Lee Z.; Ahn J.-H. Degradation behaviors and mechanisms of MoS2 crystals relevant to bioabsorbable electronics. NPG Asia Mater. 2018, 10 (8), 810–820. 10.1038/s41427-018-0078-6.

[ref32] Lyshtva P.; Voronova V.; Barbir J.; Leal Filho W.; Kröger S. D.; Witt G.; Miksch L.; Saborowski R.; Gutow L.; Frank C.; Emmerstorfer-Augustin A.; Agustin-Salazar S.; Cerruti P.; Santagata G.; Stagnaro P.; D’Arrigo C.; Vignolo M.; Krång A.-S.; Strömberg E.; Lehtinen L.; Annunen V. Degradation of a poly(3-hydroxybutyrate-co-3-hydroxyvalerate) (PHBV) compound in different environments. Heliyon 2024, 10 (3), e24770 10.1016/j.heliyon.2024.e24770. 38322905 PMC10844030

[ref33] Gross R. A.; Kalra B. Biodegradable Polymers for the Environment. Science 2002, 297 (5582), 803–807. 10.1126/science.297.5582.803. 12161646

[ref34] Zhang S.; Bristow N.; Wyn David T.; Elliott F.; O’Mahony J.; Kettle J. Development of an organic photovoltaic energy harvesting system for wireless sensor networks; application to autonomous building information management systems and optimization of OPV module sizes for future applications. Sol. Energy Mater. 2022, 236, 111550 10.1016/j.solmat.2021.111550.

[ref35] Singh J.; Kaur R.; Singh D. Energy harvesting in wireless sensor networks: A taxonomic survey. Int. J. Energy Res. 2021, 45 (1), 118–140. 10.1002/er.5816.

[ref36] Chou J. C.; Chen J. T.; Liao Y. H.; Lai C. H.; Chen R. T.; Tsai Y. L.; Lin C. Y.; Chen J. S.; Huang M. S.; Chou H. T. Wireless Sensing System for Flexible Arrayed Potentiometric Sensor Based on XBee Module. IEEE Sens. J. 2016, 16 (14), 5588–5595. 10.1109/JSEN.2016.2570285.

[ref37] Mu B.; Cao G.; Zhang L.; Zou Y.; Xiao X. Flexible wireless pH sensor system for fish monitoring. Sens. BioSensing Res. 2021, 34, 100465 10.1016/j.sbsr.2021.100465.

[ref38] Pavličková M.; Lorencová L.; Hatala M.; Kováč M.; Tkáč J.; Gemeiner P. Facile fabrication of screen-printed MoS2 electrodes for electrochemical sensing of dopamine. Sci. Rep. 2022, 12 (1), 11900 10.1038/s41598-022-16187-2. 35831476 PMC9277599

[ref39] Overly T. G. S.; Park G.; Farinholt K. M.; Farrar C. R. Development of an extremely compact impedance-based wireless sensing device. Smart Mater. Struct. 2008, 17 (6), 065011 10.1088/0964-1726/17/6/065011.

[ref40] Bristow N.; Rengaraj S.; Chadwick D. R.; Kettle J.; Jones D. L. Development of a LoRaWAN IoT Node with Ion-Selective Electrode Soil Nitrate Sensors for Precision Agriculture. Sensors 2022, 22 (23), 9100 10.3390/s22239100. 36501798 PMC9739143

[ref41] Li H.; Liu S.; Li X.; Hao R.; Wang X.; Zhang W.; Zheng Z.; Feng Q. All-Solid, Ultra-Micro, and Ultrasensitive pH Sensor by Monolayer MoS2-Based Array Field-Effect Transistors. ACS Appl. Nano Mater. 2021, 4 (9), 8950–8957. 10.1021/acsanm.1c01568.

[ref42] Jiang J.; Li H.; Dai L.; Hu H.; Zhao C. Raman scattering of 2H-MoS2 at simultaneous high temperature and high pressure (up to 600 K and 18.5 GPa). AIP Adv. 2016, 6 (3), 035214 10.1063/1.4944832.

[ref43] Gołasa K.; Grzeszczyk M.; Leszczyński P.; Faugeras C.; Nicolet A. A. L.; Wysmołek A.; Potemski M.; Babiński A. Multiphonon resonant Raman scattering in MoS2. Appl. Phys. Lett. 2014, 104 (9), 092106 10.1063/1.4867502.

[ref44] Alvarez F.; Arena M.; Auteri D.; Binaglia M.; Castoldi A. F.; Chiusolo A.; Colagiorgi A.; Colas M.; Crivellente F.; De Lentdecker C.; De Magistris I.; Egsmose M.; Fait G.; Ferilli F.; Gouliarmou V.; Nogareda L. H.; Ippolito A.; Istace F.; Jarrah S.; Kardassi D.; Kienzler A.; Lanzoni A.; Lava R.; Leuschner R.; Linguadoca A.; Lythgo C.; Magrans O.; Mangas I.; Miron I.; Molnar T.; Padovani L.; Panzarea M.; Parra Morte J. M.; Rizzuto S.; Serafimova R.; Sharp R.; Szentes C.; Szoradi A.; Terron A.; Theobald A.; Tiramani M.; Vianello G.; Villamar-Bouza L.; Peer review of the pesticide risk assessment of the active substance ethephon. EFSA J. 2023, 21 (1), e07742 10.2903/j.efsa.2023.7742. 36742463 PMC9888216

[ref45] Koch D.; Friedl A.; Mihalyi B. Influence of different LCIA methods on an exemplary scenario analysis from a process development LCA case study. Environ. Dev. Sustain. 2023, 25 (7), 6269–6293. 10.1007/s10668-022-02302-w.

