# Supplementary material for: Hybrid Agricultural Monitoring System with Detachable, Biodegradable, and Printed pH Sensors with a Recyclable Wireless Sensor Network for Sustainable Sensor Systems
Source: ACS Appl Electron Mater. 2025 Mar 21;7(7):2731–40. doi: 10.1021/acsaelm.4c02141 (PMC11984102; doi:10.1021/acsaelm.4c02141)
Supplement: Supplementary file 1 — el4c02141_si_001.pdf [file el4c02141_si_001.pdf]

Supporting Information

Hybrid agricultural monitoring system with detachable biodegradable and printed pH sensor with a recyclable wireless sensor network for sustainable sensor systems

Andrew Rollo,[1] Joseph Cameron,[1] Jose Diego Fernandes Dias,[1] Radosław Cichocki,[2] Beata Synkiewicz-Musialska,[2] Jia Ren,[3] Shoushou Zhang,[1,4]* Jeff Kettle[1]*

[1]James Watt School of Engineering, University of Glasgow, Glasgow, G12 8QQ, United Kingdom

[2]Łukasiewicz Research Network – Institute of Microelectronics and Photonics, Kraków, 30-701, Poland

[3]Electronic Information and Physics College, Central South University of Forestry and Technology, Changsha, Hunan, 410004, P. R. China

[4]Bangor College, Central South University of Forestry and Technology, Changsha, Hunan, 410004, P. R. China

Email: shoushou.zhang@glasgow.ac.uk; jeff.kettle@glasgow.ac.uk

Table of Contents

Figure/Table	Title	Page
**Table S1**	Information on elemental nutrients, present in the fabricated pH sensors	S2
**Table S2**	Range and power outputs for IoT Technologies	S3
**Figure S1**	Raman spectra of MoS_2 layer from fabricated sensor at different areas of the film	S3
**Figure S2**	Fitted circuits for the impedance of PHBV/graphene-carbon/MoS_2 sensors in different pH solutions	S4
**Figure S3**	Nyquist plots of fitted equivalents circuits for the impedance of PHBV/graphene-carbon/MoS_2 sensors in different pH solutions	S5
**Table S3**	LCA results for the WSN with an attached sensor	S5
**Table S4**	LCA results for the MoS_2 Sensor	S5
**Figure S4**	3D image of PCB board of the impedance WSN node	S6
**Figure S5**	Pin Configuration of AD5933	S8
**Figure S6**	Basic construction of the WSN node on a breadboard	S9
**Figure S7**	Schematic of WSN for impedance sensing	S10

Table S1. Information on elemental nutrients, present in the fabricated pH sensors

Nutrient Family	Nutrient	Typical concentration (%) in plant matter	Form taken up by plants	Major functions in plants	Main crops in Canada, Europe and UK (ambient soil pH range requirement level) Examples	Safe concentration in soil	Effect of excess	Effects of deficiency
**Primary**	Carbon (C)	45	Carbon dioxide (CO_2), Bicarbonate (HCO_3^-)	Plant structures	Wheat (6.4), Rice (6-7), Corn (5.8-6.2), Soybeans (5.8-6.2), Canola (5.5-7.5), Potatoes (4.5-8.5)	8.10 g/kg 0-20 cm; 6.57g/kg 20-40 cm; 2.63 g/kg 40-60 cm; 1.97 g/kg 60-80; 1.80 g/kg 80-100 [1]	Excessive carbon content is not typically a concern unless it results from excessive organic matter additions, which can cause nitrogen immobilization and nutrient imbalances.[2]	Organic carbon deficiency disrupts nutrient availability, negatively impacting plants. Low organic carbon levels reduce microbial activity, decreasing nutrient accessibility. Changes in microbial diversity affect interaction intensity. These interactions indirectly influence carbon metabolism and plant health (stunted growth and development). [3]
	Oxygen (O)	45	Water (H_2O)	Respiration, energy production, plant structures		Oxygen is not typically quantified in terms of weight content in soil, as it is primarily obtained through soil aeration and water content.	Excess oxygen (under water-deficient conditions), by stimulating oxidation processes, promotes the degradation of the organic component, it may cause damage to the plant root system and disruption of metabolic processes [4]	Root suffocation, hinder nutrient uptake, and result in poor plant growth and yellowing leaves, disturbing growth, nutrient and water uptake, and hormonal balances [5], [6]
	Hydrogen (H)	6.0	Water (H_2O)	pH regulation, water retention, synthesis of carbohydrate s		Hydrogen is primarily obtained through water and organic sources rather than being directly measured in soil analyses	Excess water, caused by soil flooding or deeper submergence, is one of the major abiotic stresses that limit plant growth, species distribution, and agricultural productivity. [7]	Drought reduces leaf size, stem extension, and root growth, disrupts water-use efficiency, impairs photosynthesis through stomatal closure and enzyme damage, increases oxidative stress causing tissue damage, and triggers adaptive mechanisms like water conservation, deep root development, osmotic adjustment, and activation of drought-responsive genes and hormones for stress tolerance. [8]
**Secondary**	Sulfur (S)	0.03	Sulfate (SO_4^{2-})	Protein, amino acid, vitamin and oil formation		17-397 µg/g in the topsoil [9]	Excess sulfur rarely harms plants, but can be energetically wasteful or lead to osmotic imbalances, plant exposed to high external sulfate affect its homeostasis [10]	Older leaf yellowing, inhibited growth, and reduced seed formation; sulphate deprivation typically shifts biomass allocation towards root production, enhancing root morphology and increasing the overall absorptive surface of the root system [10].
**Micro**	Molybdenum (Mo)	0.00001	Molybdate ions ($HMoO^{4-}$, MoO_4^{2-})	Enzyme activity and nitrogen fixation in legumes	Rice, potatoes (low pH soil)	Average concentration was reported as 1.5 mg/kg, with a range from 0.1 to 81.8 mg/kg. [11]	toxicity and nutritional imbalances in plants, (legumes) as high levels of molybdenum uptake (>30 mg/kg) can reduce the copper to molybdenum (Cu) ratio below the recommended limit, adversely affecting plant health (growth and metabolism malfunctions) [12]	Yellowing of leaves and atypical leaf growth, chlorosis, stunted stature, disrupted pollen formation. [13]

Table S2. Range and power outputs for IoT Technologies

	ZigBee	WiFi	Bluetooth	LoRa	GPRS	3G	4G	5G
**Standard**	IEEE 802.15.4	IEEE 802.11	IEEE 802.15.1	IEEE 802.15.4g	N/A	LTE	LTE	3GPP
**Frequency band**	868 MHz/915 MHz/2.4 GHz	2.4 GHz	2.4 GHz	869 MHz/915 MHz	900 – 1800 MHz	900/1800/ 2300 MHz	900/1800/ 2300 MHz	Sub-6GHz/mm wave bands
**Power consumption in Tx mode**	36.9 mW	835 mW	2.5 – 10 mW	100 mW	560 mW	32 – 200 mW	32 – 200 mW	Higher than 4G
**Data rate**	20/40/250 kbps	11-54 and 150 Mpbs	1 – 3 Mbps	50 kbps	Up to 170 kbps	5/12 Mbps	5/12 Mbps	> 1Gbps
**Communication range**	100 m	100 m	10 – 100 m	5 km	1 – 10 km	Cellular based	Cellular based	Cellular based
**Application**	WPANs, WSNs, and agriculture	WLANs	Wireless sensors	Agriculture, smart grid, environment control, lighting control	AML demand response, HAN	Video conferencing, mobile, TV, GPS, wearable devices	Video conferencing, mobile, TV, GPS, wearable devices	High resolution video streaming, remote control of vehicle, robots, medical procedures

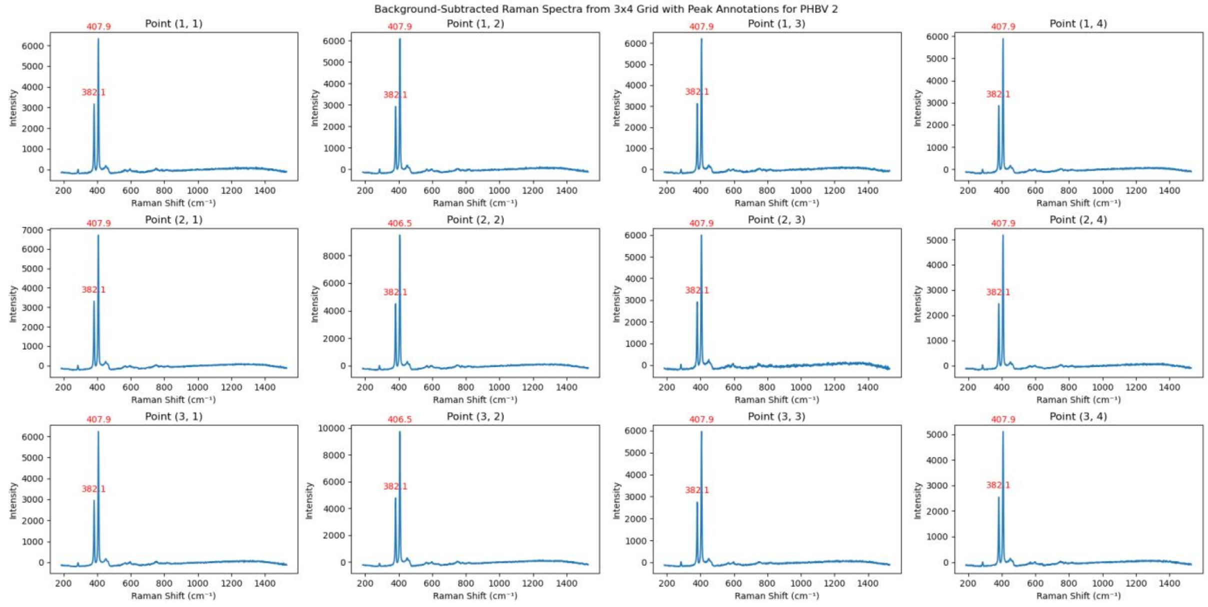

Figure S1. Raman spectra of MoS_2 layer from fabricated sensor at different areas of the film

pH 3	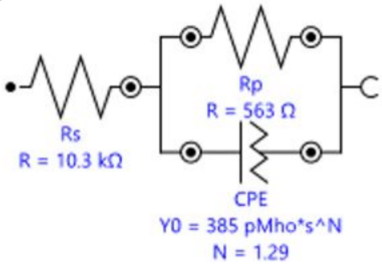
pH 4	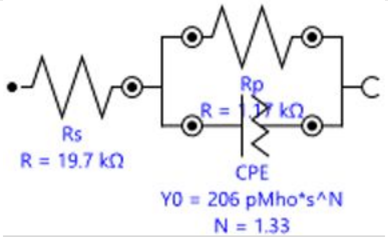
pH 5	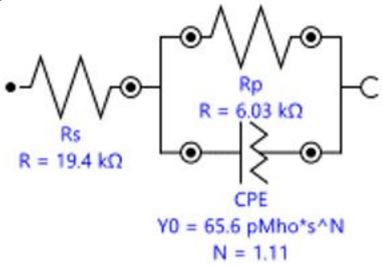
pH 6	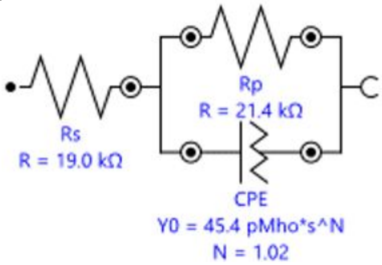
pH 7	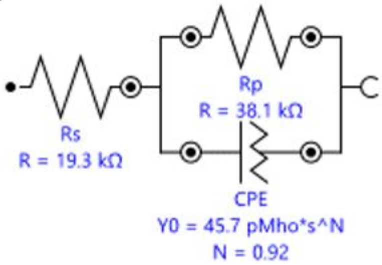
pH 8	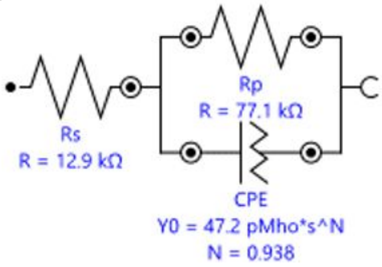

Figure S2. Fitted circuits for the impedance of PHBV/graphene-carbon/MoS_2 sensors in different pH solutions

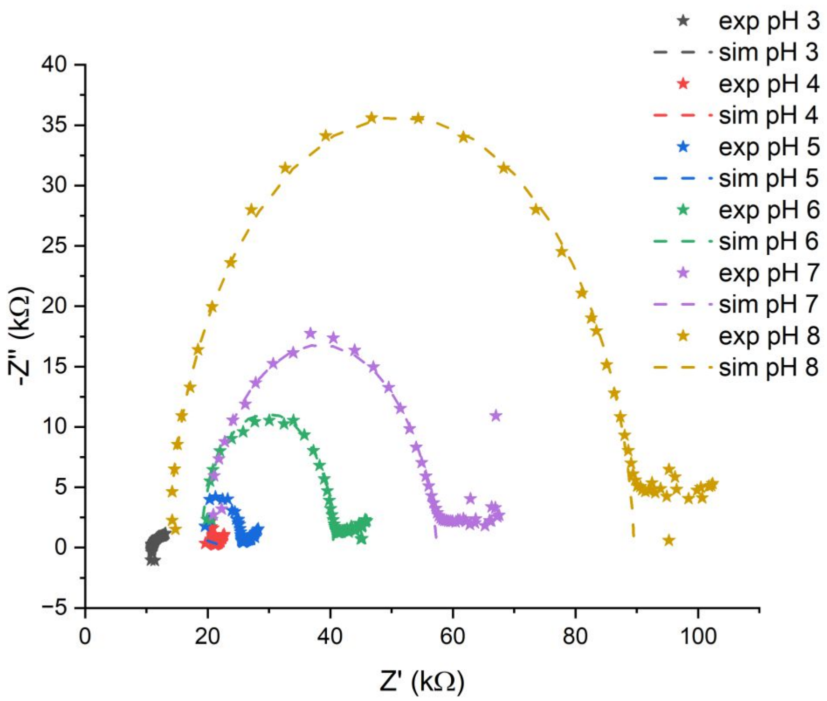

Figure S3. Nyquist plots of fitted equivalents circuits for the impedance of PHBV/graphene-carbon/MoS_2 sensors in different pH solutions

Table S3. LCA results for the WSN with an attached sensor

Category	Units	Antenna	Battery	Connectors	Electricity	ICs	Passives	PCB	Processing	Sensor	Total
**ADP elements**	kg SO_2 eq.	2.89E-07	1.87E-06	1.81E-06	4.50E-08	3.14E-04	1.74E-05	9.42E-05	2.70E-07	-6.98E-09	0.00E+00
**ADP fossil**	MJ	1.89E-01	5.42E+00	5.45E+00	1.57E+00	3.11E+01	4.09E+00	9.16E+00	6.50E-01	2.10E-01	5.78E+01
**AP**	kg SO_2 eq.	9.68E-05	1.13E-03	5.31E-04	3.20E-04	1.29E-02	8.48E-03	3.80E-03	1.19E-04	2.90E-05	2.74E-02
**EP**	kg phosphate eq.	6.05E-06	4.61E-04	2.04E-04	2.40E-05	8.86E-04	1.54E-04	3.26E-04	1.85E-05	1.52E-05	2.09E-03
**FAETP**	kg DCB eq.	1.86E-04	9.67E-04	8.20E-03	2.24E-04	5.86E-03	4.67E-03	4.77E-03	3.14E-05	3.54E-04	2.53E-02
**GWP 100 years**	kg CO_2 eq.	2.01E-02	4.30E-01	1.58E-01	1.15E-01	2.55E+00	3.35E-01	8.56E-01	2.50E-02	1.14E-02	4.50E+00
**HTP**	kg DCB eq.	1.19E-02	2.58E-02	6.70E-01	5.26E-03	3.11E-01	2.05E-01	2.49E-01	4.49E-03	1.04E-03	1.48E+00
**MAETP**	kg DCB eq.	1.98E+01	2.74E+01	2.99E+01	2.08E+01	1.47E+03	8.00E+01	1.20E+02	9.35E-01	1.50E+00	1.77+03
**ODP**	kg R-11 eq.	1.21E-10	1.79E-11	1.45E-12	3.27E-12	1.30E-11	1.37E-12	1.64E-10	1.07E-14	1.22E-10	4.44E-10
**POCP**	kg ethene eq.	7.68E-06	8.87E-05	6.44E-05	1.85E-05	7.92E-04	3.72E-04	3.20E-04	1.18E-05	3.16E-04	1.99E-03
**TETP**	kg DCB eq.	4.53E-04	6.45E-04	4.34E-04	2.65E-04	3.25E-03	2.15E-03	3.99E-03	4.28E-05	6.04E-05	1.13E-02

Table S4. LCA results for the MoS_2 Sensor

Category	Units	Conductive ink	Electricity	MoS_2 paste	Processing	Substrate	Total
**ADP elements**	kg SO_2 eq.	5.82E-12	2.29E-09	2.15E-08	1.92E-09	-3.27E-08	-6.98E-09
**ADP fossil**	MJ	1.53E-04	8.02E-02	7.69E-05	1.13E-01	1.73E-02	2.10E-01
**AP**	kg SO_2 eq.	1.07E-08	1.63E-05	5.16E-08	2.94E-06	9.66E-06	2.90E-05
**EP**	kg phosphate eq.	1.28E-09	1.22E-06	6.90E-09	7.61E-07	1.32E-05	1.52E-05
**FAETP**	kg DCB eq.	5.45E-08	1.14E-05	7.98E-08	1.45E-04	1.97E-04	3.54E-04
**GWP 100 years**	kg CO_2 eq.	4.96E-06	5.85E-03	5.49E-06	2.40E-03	3.16E-03	1.14E-02
**HTP**	kg DCB eq.	2.32E-07	2.68E-04	9.11E-07	2.61E-04	5.11E-04	1.04E-03
**MAETP**	kg DCB eq.	2.85E-04	1.06E+00	6.14E-04	1.39E-01	3.01E-01	1.50E+00
**ODP**	kg R-11 eq.	3.92E-17	1.67E-13	4.93E-15	1.61E-14	1.22E-10	1.22E-10
**POCP**	kg ethene eq.	2.15E-09	9.42E-07	2.65E-09	3.15E-04	7.07E-07	3.16E-04
**TETP**	kg DCB eq.	1.55E-08	1.35E-05	1.48E-08	2.57E-05	2.11E-05	6.04E-05

Further details of the WSN circuitry and performance

The Wireless Sensor Network (WSN), see figure 1, node is an integration of components designed for effective impedance monitoring and data transmission. Central to its operation is a microcontroller (MCU), which in this case is the ATMega328P, combined with a battery, energy management module, sensors, and a wireless transmission module. This setup ensures the reliable collection and transfer of data related to parameters such as humidity, temperature, light levels, time, and impedance. The developed sensor node mainly contains three blocks; the ATmega328P, which is used as the control unit, sensors including temperature sensor, lux sensor, real time clock module and the impedance module, and finally, the communication module, which is also constructed using a ZigBee module and protocol. A 3D image of the PCB of the WSN node is shown in figure S4.

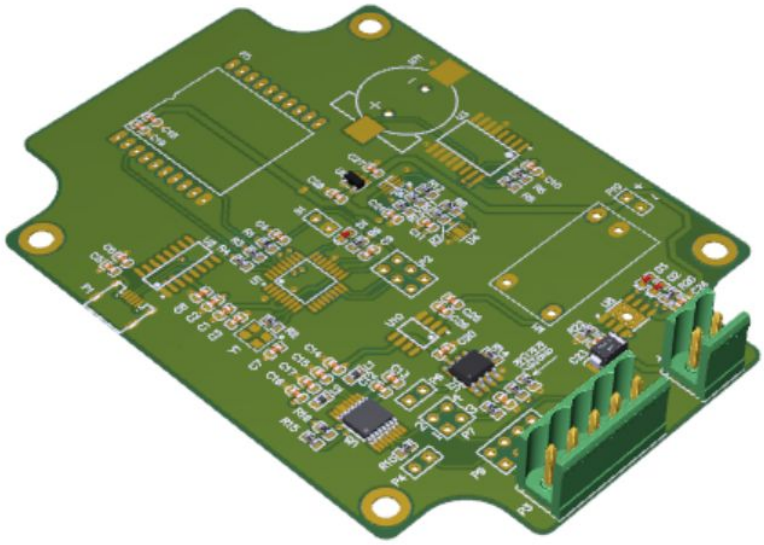

Figure S4. 3D image of PCB of the impedance WSN node

The ATMega328P is a popular choice in embedded systems due to its flexibility across diverse applications such as robotics, smart home technologies, and industrial control systems. Built on advanced RISC architecture, this 8-bit microcontroller provides 32 KB of ISP flash memory with self-programming capabilities, 1KB EEPROM for data retention during power outages, and 2KB SRAM for efficient processing. Its comprehensive features make it an ideal control unit for WSNs, enabling seamless integration and management of sensors and communication modules. One of the key features of the ATMega328P is its 10-bit ADC (Analog-to-Digital Converter), supporting up to eight channels for converting analog signals into digital formats, making it useful in various applications. Its robust communication

functionality includes support for UART (Universal Asynchronous Receiver-Transmitter), SPI (Serial Peripheral Interface), and I2C (Inter-Integrated Circuit) protocols, enabling seamless interfacing with sensors and other devices.

The operating voltage range of the ATMega328P chip is between 1.8 V to 5.5 V, and it works within a temperature range of -40 to 85°C. The ATMega328P is known for its low power consumption, ideal for energy-efficient and battery-powered devices

Data transmission within the WSN relies on XBee S2C chips that operate using the ZigBee communication protocol. ZigBee is recognized for its low power consumption, cost efficiency, and real-time monitoring capability, making it a preferred choice across industries including smart agriculture, health monitoring, and environmental systems. Operating on IEEE 802.15.4, ZigBee supports transmission frequencies such as 2.4 GHz, 868 MHz, and 915 MHz, with indoor ranges of 30–50 meters and outdoor ranges of 100–200 meters, which can be enhanced by mesh networking. The network in this project employs a star topology, where all nodes are connected to a central coordinator. This configuration simplifies installation and operation, especially in small-scale applications, while ensuring reliable data transmission.

To power the WSN nodes sustainably, organic photovoltaic (OPV) cells from InfinityPV, Denmark, are employed. These customizable solar panels enable energy harvesting, addressing varying power requirements of the WSN. Machine learning methods have been used to analyze the performance of the nodes, optimizing the size and balance between solar panels, batteries, and the system load. This energy-efficient design reduces reliance on conventional power sources and enhances the system's overall scalability and cost-effectiveness.

The core components of the WSN nodes are organized into three main blocks. The first is the microcontroller unit, represented by the ATMega328P, which orchestrates the system's functions. Second, an array of advanced sensors measures environmental variables and system parameters. These include the SHT31 for humidity and temperature (providing precise measurements with ±2% accuracy for humidity and ±0.3°C for temperature), the TSL2591 light sensor (capable of detecting 0.0017 Lux to 88,000 Lux), and the DS3231 real-time clock module for accurate timekeeping with battery backup. Additionally, the AD5933 impedance converter and ADR435 IC ensure accurate analysis and voltage management for specialized applications. The third block involves the ZigBee communication module, which seamlessly transmits collected data for analysis and decision-making.

The SHT31 integrates both humidity and temperature sensing into a compact, reliable device suitable for real-time environmental monitoring. Its capacity to span the entire range of

relative humidity and broad temperature limits make it particularly adaptable to varied conditions. Similarly, the TSL2591 sensor stands out for its extensive sensitivity and low power consumption. With the ability to differentiate between visible and infrared light, it is highly effective in applications that require detailed light analysis. The DS3231 enhances the WSN node with its robust timing capabilities, featuring a crystal oscillator for consistent operation across diverse power states.

The AD5933 impedance converter significantly expands the analytical capabilities of the WSN nodes. With its built-in 12-bit ADC and 16 MHz frequency generator, it supports frequency sweeps from 1 Hz to 100 kHz and performs 1024-point Discrete Fourier Transform (DFT) processing onboard. This enables detailed analysis of impedance properties, providing phase and magnitude information essential for electrochemical studies. Calibration of the device using a reference resistor ensures accurate impedance measurements, bolstering its reliability in sophisticated sensing tasks. Based upon our measurements and the AD5933 IC, we estimate resolution at 0.01 Ω at 1 kHz. Calibration of the AD5933 is achieved using a feedback resistor, whose value should closely match the impedance being measured. One of the key features of the AD5933 module is its ability to measure complex impedance, providing both magnitude and phase values. This capability is particularly valuable for analysing the

electrochemical properties of various materials or systems from multiple perspectives.

The Pin Configuration of AD5933 is shown in Figure S5. As shown in the configuration, the feedback resistor is connected between the fourth and fifth pins of the AD5933 chip. The measurement range of the impedance values could be affected by the value of the feedback resistor.

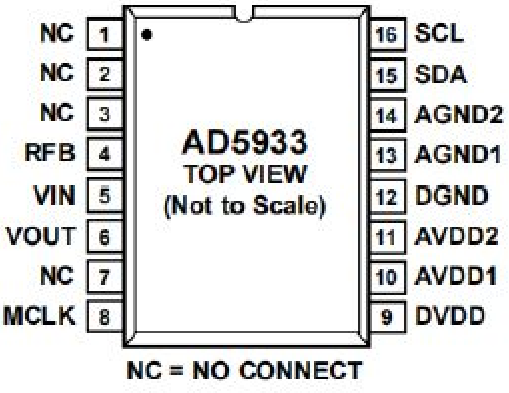

Figure S5. Pin Configuration of AD5933

Energy management is achieved through the integration of lithium polymer batteries recharged via OPV modules, augmented by the CN3163 chip. The CN3163 chip handles power management efficiently, utilizing synchronous rectification and low-dropout voltage regulation to optimize energy conversion. This combination not only supports the sustainability goals of the WSN but also ensures uninterrupted operation even in variable environmental conditions.

The development of this WSN node highlights the flexibility and utility of combining versatile components into an integrated system capable of delivering accurate data and reliable communication provides application in impedance sensing underscores its adaptability, while features like scalable solar energy systems and advanced sensing modules position it as a forward-thinking solution for modern monitoring and analysis needs. By leveraging robust microcontrollers, effective communication protocols, and sustainable energy sources, this design establishes a benchmark for future innovations in WSN technology. Shown in Figure S6 is the construction on a breadboard and Figure S7 shows a schematic of the WSN for impedance sensing

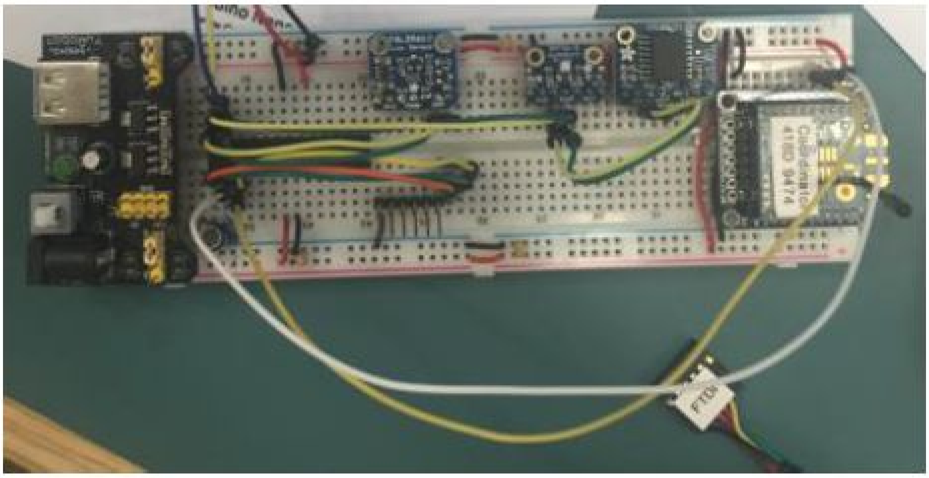

Figure S6. Basic construction of the WSN node on a breadboard

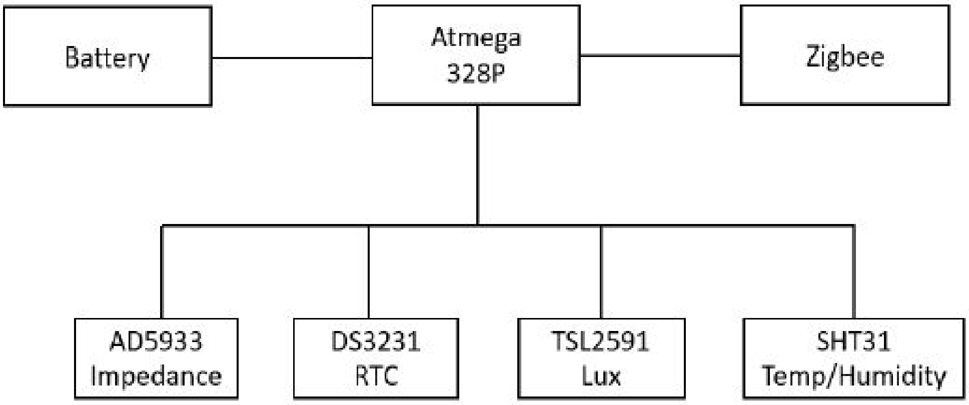

Figure S7. Schematic of WSN for impedance sensing

References

1. Nelson, P. N.; Dictor, M.C.; and Soulas G, Availability of organic carbon in soluble and particle-size fractions from a soil profile. Soil Biology and Biochemistry. **1994**, 26 (11), 1549-1555. DOI: 10.1016/0038-0717(94)90097-3
2. Jansson, S. L.; and Persson, J., Mineralization and immobilization of soil nitrogen. Nitrogen in agricultural soils. Wiley, **1982**, 229-252.
3. Gao, G.; Li G.; Liu, M.; Liu, J.; Ma, S.; Li, D.; Liang, X.; Wu, M., Li, Z., Microbial carbon metabolic activity and bacterial cross-profile network in paddy soils of different fertility. Applied Soil Ecology. **2024**, 195, 105233. DOI: 10.1016/j.apsoil.2023.105233
4. Ostrowski, J.; Sławiński C.; Walczak R., Evaluation and cartographic presentation of arable soil susceptibility to hydrooxygenic degradation. Woda-Środowisko-Obszary Wiejskie. **2004**, 4, 185-200.
5. Drew, M. C., Plant injury and adaptation to oxygen deficiency in the root environment: A review. Plant and soil **1983**, 75(2), 179-199. DOI: 10.1007/BF02375564
6. Armstrong, W.; Drew M.C., Root growth and metabolism under oxygen deficiency. Plant roots. CRC Press, **2002**, 1139-1187.
7. Jackson, M. B.; and Colmer T.D, Response and adaptation by plants to flooding stress. Annals of botany **2005**, 96 (4) 501-505. DOI: 10.1093/aob/mci205
8. Farooq, M.; Wahid, A.; Kobayashi, N.; Fujita, D.; Basra, S.M.A., Plant drought stress: effects, mechanisms and management. Sustainable agriculture. **2009**, 153-188. DOI: 10.1051/agro:2008021
9. Wang, S. P.; Wang, Y. F.; Chen, Z. Z.; Schnug, E.; Haneklaus, S., Sulphur concentration of soils and plants and its requirement for ruminants in the Inner Mongolia steppe of China. Grass Forage Sci. **2001**, 56 (4), 418-422. DOI: 10.1046/j.1365-2494.2001.00285.x
10. Hawkesford, M.J.; Luit J. De Kok, L.J., Managing sulphur metabolism in plants. Plant Cell Environ. **2006**, 29 (3), 382-395. DOI: 10.1111/j.1365-3040.2005.01470.x
11. Axelson, U.; Söderström, M.; Jonsson, A., Risk assessment of high concentrations of molybdenum in forage. Environmental geochemistry and health. **2018**, 40, 2685-2694. DOI: 10.1007/s10653-018-0132-x
12. McBride, M. B.; Richards, B.K.; Steenhuis, T.S.; Spiers, G.A., Molybdenum uptake by forage crops grown on sewage sludge-amended soils in the field and greenhouse. American Society of Agronomy, Crop Science

Society of America, and Soil Science Society of America. **2000**, 29 (3). DOI: 10.2134/jeq2000.00472425002900030021x

13. Gupta, U. C., Molybdenum in agriculture. Cambridge University Press. **1997**. DOI: 10.1017/CBO9780511574689
